# Exploring the Pro-Phagocytic and Anti-Inflammatory Functions of PACAP and VIP in Microglia: Implications for Multiple Sclerosis

**DOI:** 10.3390/ijms23094788

**Published:** 2022-04-26

**Authors:** Margo I. Jansen, Sarah Thomas Broome, Alessandro Castorina

**Affiliations:** Laboratory of Cellular and Molecular Neuroscience (LCMN), School of Life Sciences, Faculty of Science, University of Technology Sydney, Sydney, NSW 2007, Australia; margo.jansen@student.uts.edu.au (M.I.J.); sarah.j.thomasbroome@student.uts.edu.au (S.T.B.)

**Keywords:** pituitary adenylate cyclase-activating polypeptide (PACAP), vasoactive intestinal peptide (VIP), microglia, phagocytosis, anti-inflammatory, multiple sclerosis, inflammation

## Abstract

Multiple sclerosis (MS) is a chronic neuroinflammatory and demyelinating disease of the central nervous system (CNS), characterised by the infiltration of peripheral immune cells, multifocal white-matter lesions, and neurodegeneration. In recent years, microglia have emerged as key contributors to MS pathology, acting as scavengers of toxic myelin/cell debris and modulating the inflammatory microenvironment to promote myelin repair. In this review, we explore the role of two neuropeptides, pituitary adenylate cyclase-activating polypeptide (PACAP) and vasoactive intestinal peptide (VIP), as important regulators of microglial functioning during demyelination, myelin phagocytosis, and remyelination, emphasising the potential of these neuropeptides as therapeutic targets for the treatment of MS.

## 1. Introduction

Multiple sclerosis (MS) is a debilitating disease affecting about 2.8 million people globally [1]. The disease is characterised by myelin damage due to an abnormal attack by immune cells, resulting in the appearance of white-matter lesions in the central nervous system (CNS), neurodegeneration, and focal neuroinflammation, which can lead to physical and neurological deficits [2,3,4,5]. There is currently no cure for MS, and the existing treatment options for MS patients primarily target the dysfunctional immune response, mainly by promoting the segregation of immune cells in peripheral compartments, thereby reducing the overall autoimmune damage to the CNS [6]. 

Microglia, the resident macrophages of the CNS, have emerged as key players in MS pathogenesis, with growing research interest focused on unveiling new mechanisms to modulate some of its biological functions [7]. In MS, microglia demonstrate dichotomous profiles, as they exert either detrimental or beneficial effects [8,9,10,11]. Microglia are by nature immune modulatory cells and respond with complex activation patterns to stress signals, by facilitating the release of pro- and anti-inflammatory cytokines and chemokines, as well as by providing trophic support to neurons and/or scavenging toxic debris through phagocytosis [12,13].

In this review, we explore the role of microglia in MS, with specific emphasis on recent findings, highlighting the critical role of these glial cells in myelin debris clearance and anti-inflammatory properties in the CNS. More specifically, this work aims to highlight the role of two neuropeptides, pituitary adenylate cyclase-activating polypeptide (PACAP) and vasoactive intestinal peptide (VIP), as key regulators of microglial responses during CNS demyelination, portraying the potential of these molecules to become therapeutic targets for MS.

## 2. Multiple Sclerosis 

### 2.1. Autoimmunity in MS

MS is a complex, multifactorial disease with unknown origin, although bulk evidence suggests that disease onset might be triggered by a combination of contributing risk factors such as certain lifestyle habits (i.e., smoking), diet, genetic, and environmental causes [14,15]. The exact pathogenesis of MS is also yet to be determined; however, it is widely accepted that most of the pathological events in MS patients are consequent to the infiltration of autoreactive peripheral immune cells into the CNS, with auto-reactive T cells being pivotal in promoting central demyelination [2,5,16]. Auto-reactive T lymphocytes are recruited to the CNS and cross the disrupted blood–brain barrier (BBB). These immune cells—mostly CD4^+^ and CD8^+^ T-cells—are believed to express myelin-specific surface antigens; hence, they are able to target myelin and oligodendrocytes (OLs), the myelin-producing cells of the CNS, causing demyelination and OL depletion [16,17,18,19]. Demyelination results in the generation of myelin debris which in turn can get oxidised, creating oxidised phosphatidylcholines (OxPCs), here referred to as toxic myelin debris, which can have detrimental effects on cellular functioning [20,21,22]. When this occurs, microglia attempt to clean “the aftermath” by engulfing the toxic myelin debris [9,23,24]. Under these inflammatory conditions, these microglia are also believed to further stimulate the activation of the infiltrated immune cells due to their antigen-presenting cell (APC) properties and via the release of inflammatory factors. This activates a vicious cycle that leads to further infiltration of peripheral immune cells (CD4^+^ and CD8^+^ T-cells, macrophages, B-cells) and damage to the CNS white matter [25,26,27]. Altogether, these data pinpoint how the uncoordinated healing response of resident glia to an immune-mediated myelin attack can exacerbate the autoimmune response, leading to the formation (or expansion) of white-matter lesions.

However, it is important to note that most of the data concerning the involvement of autoimmunity in MS have been gathered from preclinical models, such as the experimental autoimmune encephalomyelitis (EAE) mouse model of MS. The EAE model is broadly used to model MS since it mimics certain aspects of the pathology, particularly its inflammatory component [28]. However, there are some essential differences with human MS, such as the lymphocyte subpopulations that are predominantly found in EAE vs. MS (CD4^+^ T-cells vs. CD8^+^ T-cells, respectively), as well as the limited demyelination seen in these mice [28,29,30]. Nonetheless, although the model may not recapitulate the entire spectrum of pathological changes seen in MS, the EAE model has helped the scientific community gain essential insights into the role of autoimmunity and neuroinflammation in MS.

### 2.2. Demyelination in MS

Another major pathological hallmark of MS is the progressive loss of myelin in the CNS and OL cell loss. Myelin is necessary to provide insulation to axons in the brain and spinal cord, allowing proper conduction of electric impulses along the axons [31]. In healthy conditions, CNS myelin is produced by mature/fully differentiated OLs, whereas OL precursor cells (OPCs) are a proliferative group of progenitor cells that can differentiate into mature OLs, allowing both adaptive myelination and the regeneration of myelin following injury or disease [32,33]. During MS, in addition to myelin loss, there is a remarkable depletion in the pool of both OPCs and mature OLs, which hinders the process of remyelination, the main cause of axonopathy and consequent neuronal loss [11,34,35,36]. In fact, it is the resulting neurodegeneration and the increased lesion load, not demyelination (at least not directly), that correlates with the severity of disease-related disability [37,38].

Neurodegeneration in MS is not solely linked to myelin loss; it is also exacerbated by the hyperinflammatory state of the CNS [39,40]. However, the notion that neurodegeneration in MS is always preceded by inflammation has been challenged in the field [41,42]. Since limited remyelination in the MS brain is considered a major contributor to lesion development, a “hot topic” in recent years has been to elucidate the molecular mechanisms underlying the de- and remyelination process within the CNS [43,44,45]. Both processes are often studied in mice using the cuprizone model. By feeding mice a diet supplemented with 0.2–0.3% of the copper chelator cuprizone (oxalic acid bis (cyclohexylidene hydrazide)) for more than 3 weeks, remarkable oligodendrocyte death and subsequent demyelination of the CNS can be induced in a reversible manner, allowing the study of the de- and remyelination process of the brain [46].

### 2.3. Microglia in MS 

#### 2.3.1. Role of Microglia in MS

MS is characterised by both demyelination and extensive neuroinflammation, although the causal relationship between these two has yet to be determined. A crucial cell type involved in determining the fate of both processes is the microglia. In the CNS, microglia constantly survey the surrounding microenvironment, allowing for a rapid response upon the detection of a certain stressor (e.g., exposure to pathogen-associated molecular patterns (PAMPs) or damage-associated molecular patterns (DAMPs)) [12,13,47,48]. Initially, the activation pattern of microglia was thought to be compartmentalised into two distinct activated phenotypes; M1 microglia, characterised by cells releasing proinflammatory cytokines such as tumour necrosis factor-α (TNF-α), interleukin-1 (IL-1), IL-6, and IL-18, and M2 microglia, in which cells predominantly release anti-inflammatory cytokines (IL-4, IL-10, IL-13, and TNF-β) [48,49]. However, this neat subdivision of microglia phenotypes, although still in use, is progressively losing traction as more discoveries highlight the broad spectrum of dynamic temporal and spatial changes these cells undergo when stimulated [12,47]. 

In EAE mice, microglia activation promotes disease progression, as well as reduces remyelination in interferon (IFN)-γ^+^ lipopolysaccharide (LPS)-induced demyelination [50,51,52]. In the MS brain, microglial cells migrate to active lesion sites, where they represent the bulk of the initial pool of myeloid cells found around these lesions and display a typical proinflammatory phenotype [10,53,54,55]. Here, microglia act as the main APCs and show increased expression of major histocompatibility complex (MHC) I and II, CD40, CD80, and CD86, contributing to the initiation and stimulation of T-cell activation [56,57,58]. In addition, they chemotactically recruit peripheral immune cells to the lesion sites through the release of chemokines such as CCL2, CXCL3, CCL12, and CCL13 [59,60,61].

In contrast to the detrimental roles attributed to microglia during the active stages of MS (when there is a flare-up in the activity of immune cells against myelin), these cells also exert several beneficial functions during the stage of immune quiescence. Hence, it is critical to differentiate this division, as microglial cells could become important cellular targets for the development of future MS therapies. For instance, microglia have been shown to mediate immunomodulation, as well as the promotion of neuronal and oligodendrocyte repair [9,62]. In fact, there is evidence suggesting that MS disease-associated microglia found surrounding demyelinating lesions slowly transition from a proinflammatory to more anti-inflammatory phenotype as the lesion becomes less inflamed, suggesting a controlled response to demyelination that aims for repair [55,63]. Microglia also promote the proliferation and differentiation of OPCs and neuronal progenitor cells via the release of certain trophic factors, such as brain-derived neurotrophic factor (BDNF) and transforming growth factor β (TGF-β) [8,64,65,66,67].

Another critical function of microglia that is purportedly beneficial during MS is phagocytosis. Phagocytosis is the process via which cells engulf and digest large particles and debris in an attempt to maintain homeostasis [68]. Phagocytosis plays an important role in the brain, where it is known to be involved in synapse elimination, clearance of dying cells, and preventing an overflow of proinflammatory and/or anti-inflammatory cytokines [69,70]. Some research groups have reported that myelin phagocytosis could contribute to CNS demyelination [71,72]; however, there is stronger evidence, in vitro and in vivo, in support of the beneficial roles of microglia in the phagocytosis of damaged myelin in the CNS of MS patients [73,74]. Myelin-containing microglia and macrophages are found surrounding both active and chronic active lesions in MS patients [75]. Engulfment of myelin by microglia aims at facilitating the remyelination process by promoting the clearance of toxic myelin debris, whose accumulation around the damaged site prevents OPCs proliferation, thus impeding their ability to repopulate the depleted pool of OLs [70,76,77]. However, over time, the CNS of MS patients becomes unable to remyelinate lesions. Whether this depends on the reduced phagocytic activity of microglia, the hindered OPC proliferation, and/or OL maturation or their combination is still under scrutiny [11,43,44,78]. Nonetheless, it is clear why understanding which molecular mechanisms can reinstate remyelination is a focal point in MS research [45].

#### 2.3.2. Phagocytic Activity of Microglia in the CNS White Matter in MS 

Phagocytosis plays an important role in the CNS, where it is known to be involved in synapse elimination, clearance of dying cells, and the control of proinflammatory and/or anti-inflammatory cytokines in the brain [69,70].

Evidence has suggested that myelin itself is able to regulate its own phagocytic fate by balancing pro- and antiphagocytic signalling. On the one hand, myelin phagocytosis is believed to be stimulated through the activation of certain receptors including, the complement-receptor 3 (CR3), scavenger receptor-AI/II (SRA), FCγ receptor, tyrosine kinase receptor MerTK, CD36, and triggering receptor expressed by myeloid cells 2 (TREM2) [79,80,81,82,83,84,85,86]. On the other hand, myelin was shown to downregulate myelin phagocytosis through CD47 and signal regulatory protein-a (SIRP-a) signalling [87]. In MS, the expression of phagocytosis-associated receptors (also known as scavenger receptors) such as SRA and Fcg was found to be upregulated in lesions [88].

CR3 both activates and inhibits myelin phagocytosis in myeloid cells [89]. Myelin-mediated activation of CR3 signals F-actin/myosin contraction through Galectin-3 (Gal3)/K-RAS signalling, retracting the protruding filopodia, a signalling pathway that is also stimulated by SRA receptor activation [90]. CR3 activation also phosphorylates spleen tyrosine kinase (Syk), allowing for the downstream phosphorylation of cofilin (an actin-binding protein that regulates filament dynamics and depolymerisation), remodulating F-actin stabilisation and facilitating phagocytosis [89,91,92]. The FCγ receptor contributes to this process by also allowing for the activation of Syk, stimulating the Syk/cofilin/F-actin mediated engulfment of myelin [93]. 

MERTK, the gene encoding for the merTK receptor, has been described as a MS susceptibility gene, and substantial evidence supports its regulatory role in myelin phagocytosis by microglia [94,95,96,97]. However, the exact mechanism via which this occurs is still unclear. Two known ligands of merTK, Protein S and Gas6, have been found to tether the binding of apoptotic cells to merTK via binding to the phospholipid phosphatidylserine [79,98,99]. This interaction is an important “eat me” signal in myeloid cells, which triggers phagocytosis [100]. Given how Gal6 and Protein S recognise phospholipids, added to the fact that degenerating myelin results in the accumulation of phospholipids at the damaged site, it can be hypothesised that merTK may bind to these myelin-derived phospholipids directly and contribute to phagocytic signalling. However, this mechanism is still hypothetical, as MerTK signalling could also be instigated indirectly by other unknown signals yet to be unveiled. 

There is increasing interest toward comprehending the role of TREM2 and its involvement in lipid metabolism, especially in the field of MS research [86,101,102,103,104]. As the name suggests, TREM2 is expressed by immune cells, including infiltrating macrophages and resident microglia [105,106]. Although no direct risk factors for TREM2 and MS have been described, mutations in this receptor and its signalling partners are linked to the rare Nasu–Hakola disease, a disease characterised by frontal lobe dementia, CNS white-matter lesions, and widespread microglia activation [107,108,109]. In the CSF of MS patients, there are increased levels of soluble TREM2 [110]. Moreover, TREM2 expression is increased in the microglia and other phagocytes surrounding MS lesions [21,101].

TREM2 has been implicated in mediating microglial-mediated myelin clearance in MS, although it also plays a role in amyloid-β and apoptotic cell clearance, suggesting a possible involvement in Alzheimer’s disease pathology [111]. TREM2 binds directly to phospholipids, triggering phagocytic activity in microglia through its interaction with the DNAX-activating protein of 12 kDa (DAP12) [84,85,86,111]. Activated TREM2 receptors have been shown to phosphorylate Syk through DAP12, triggering the stimulation of F-actin polymerisation, as highlighted above. 

Following cuprizone-induced demyelination, TREM2^−/−^ mice show reduced myelin phagocytosis and increased myelin degradation, two phenomena that in part are believed to be caused by the inability of microglia to activate lipid capture and lipid metabolism pathways [86,112]. This inability to metabolise myelin debris has been studied in detail in a recent study by Nugent et al. (2020), where TREM2^−/−^ microglia were shown to pathologically accumulate myelin-derived cholesteryl ester lipids [103]. In the EAE model of MS, blocking TREM2 activity during the effector stage of the EAE model caused more severe pathological response and resulted in more diffuse demyelination pattern [113]. In fact, TREM2 is required to resolve the innate immune response upon detection of myelin debris [104]. Additionally, single-cell RNA sequencing of lesioned spinal cords demonstrated that, following injections of oxidised phospholipids (OxPCs)—common end-products found in MS lesions—into the mouse CNS, only microglia expressing high levels of TREM2 were responsive to OxPCs [21,114]. TREM2^−/−^ mice had a more severe demyelinating response to OxPC injection compared to wildtype mice. In line with these findings, overexpression of TREM2 receptors seems to increase the phagocytic activity of microglia and reduces the release of proinflammatory cytokines [102,115]. Additionally, direct activation of TREM2 on microglia using antibodies was shown to promote myelin clearance, as well as OL recruitment and maturation [101]. 

**Figure 1:**
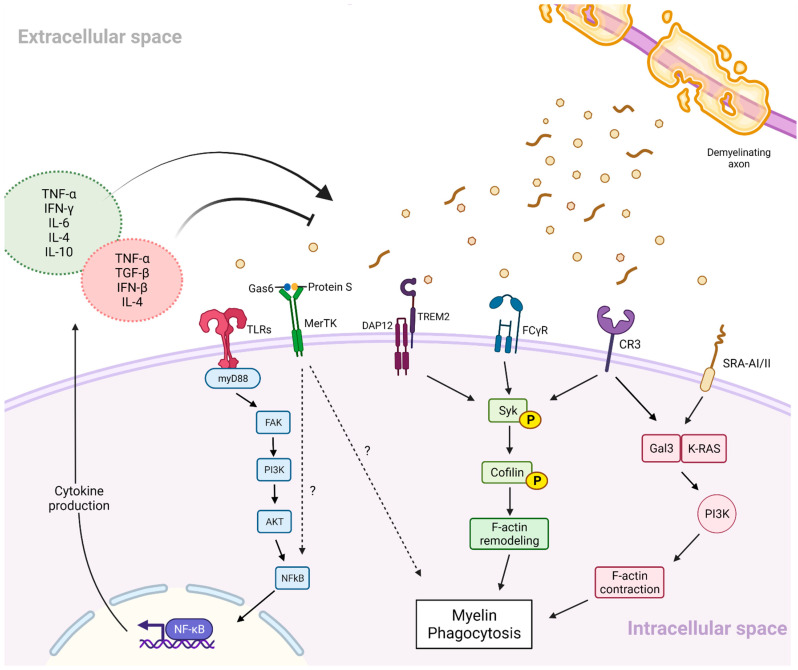
**Schematic overview of the main signalling pathways involved in myelin phagocytosis by microglia**. In the figure, the molecular signalling pathways associated with microglial-mediated myelin debris phagocytosis are shown. Briefly, myelin debris is recognised by TLRs, MerTK, TREM2, FCγR, CR3, or SRA-AI/II receptors, which in turn stimulates the release of pro- and anti-inflammatory cytokines. Cytokines released in the extracellular space trigger the activation of several signalling pathways involved in the remodelling and contraction of the cytoskeleton, thereby facilitating the engulfment of myelin debris.

In conclusion, the signalling mechanisms contributing to myelin phagocytosis and the engulfment of other lipid-rich debris are complex and greatly overlapping. For clarity purposes, these mechanisms are summarised in Figure 1. Furthermore, it should be highlighted that these signalling pathways also contribute to other important microglial functions, some of which are explored in the next section. Understanding how these pathways control microglial responses to toxic myelin debris and regulate their metabolism may be of fundamental importance for the future development of therapeutic targets to treat demyelinating diseases, including MS.

#### 2.3.3. Inflammatory Signalling and Myelin Phagocytosis

Although phagocytosis can be triggered through the activation of lipid-sensing receptors expressed in the cell surface of microglia (Figure 1), trophic factors and/or inflammatory mediators can also activate this biological process. A selection of these factors includes TNF-α, transforming growth factor β1 (TGF-β1), Activin-A, and Gal3, as well as a range of inflammatory mediators that are secreted by lesion-associated microglial cells, and whose release also promotes OPC recruitment, proliferation, and differentiation [66,116,117,118,119,120]. However, how these factors released by microglia can self-regulate myelin phagocytosis is a relatively understudied topic. Below, the most recent highlights from the literature are provided.

Myelin was found to stimulate cytokine expression and release through the activation of focal adhesion kinase (FAK)/PI3K/Akt/NFκB signalling in a process dependent on Toll-like receptor (TLR)/myeloid differentiation primary response 88 (MyD88) [121,122,123,124]. This myelin-activated signalling pathway is critical for the facilitation of myelin phagocytosis. Specifically, previous evidence has shown that myelin induced the expression of TNF-α, IFN-γ, IL-1β, IL-6, IL-12, IL-10, IL-17, macrophage migration inhibitory factor (MIF), matrix metalloproteinase 9 (MMP9), C–X–C motif chemokine ligand 10 (Cxcl10), and chemokine (C–C motif) ligands 3 and 4 (Ccl3 and Ccl4), whereas it downregulated TGF-β and IL-4 levels in bone marrow-derived macrophages [96,124]. This process appears to be partly regulated by the CR3 receptor. In fact, treatment with TNF-α but not IL-1 reduced the amount of myelin ingested by macrophages via their complement receptor type 3 (CR3), suggesting a regulatory role of this cytokine in CR3-dependent myelin phagocytosis [125]. However, it is noteworthy that there are indications where TNF-α, TGF-β, and IFN-β can stimulate phagocytosis in a CR3-independent manner [79,121,122,126]. Since TNF-α is also an important signalling molecule in apoptosis, it is important that TNF-α-induced stimulation of myelin phagocytosis does not result in excessive phagocyte death [127]. Therefore, an important role of TGF-β is also to prevent apoptosis of myelin-laden microglia by limiting TNF-α expression and lowering oxidative stress [128]. Moreover, blocking IL-6 signalling using anti-IL-6 receptor antibodies increased the expression of phagocytic markers, which coincided with a reduction in tissue damage and accumulation of myelin debris, suggesting a regulating role of this cytokine in microglial phagocytosis [129]. On the other hand, contradicting evidence has been published regarding the possible role of IL-4 in (myelin) phagocytosis. In fact, some studies have reported that IL-4 treatment reduced the expression of phagocytosis-related genes in microglia and dampened phagocytosis [121,130], whereas others have indicated that both IL-4 and IL-10 stimulated phagocytosis in cultured microglia, likely through the upregulation of TREM2 [131,132]. It is possible that the discrepancy can be explained by the different experimental approaches used. Nonetheless, it should be highlighted that other reports have found that the upregulation of IL-10 is lost in MS patient-derived macrophages, along with a decline in phagocytic capacity, which is restored after TGF-β treatment [97]. 

The V-type immunoglobulin domain-containing suppressor of T-cell activation (VISTA) receptor is a known regulator of cytokine production (such as IFN-γ, TNF-α, and IL-17) and suppresses CD4^+^ and CD8^+^ T-cell activation [133,134]. Evidence from VISTA KO microglia demonstrated a reduction in myelin phagocytosis [135]. Moreover, VISTA levels appear to be reduced in the EAE model, as well as MS patient active and chronic active lesions, but not in the normal-appearing white matter [135,136], further supporting a role of inflammatory mediators in regulating microglial phagocytic activity.

According to the data reported, it seems plausible that manipulating pathways that regulate the release of inflammatory mediators with known prophagocytic effects may lead to the shift of microglia (and perhaps of other myeloid cells) towards a phenotype that is more prone to “clear” toxic myelin debris.

## 3. PACAP and VIP

The neuropeptides pituitary adenylate cyclase-activating polypeptide (PACAP) and vasoactive intestinal peptide (VIP) are widely distributed throughout the central and peripheral nervous systems, where they exert pleiotropic neuroprotective and immunomodulatory activities [137]. In view of their therapeutic potential, these naturally occurring peptides have been linked to a range of neurodegenerative and neuroinflammatory diseases [138], including Parkinson’s disease [139], cognitive and mood disorders [140], and MS [141]. PACAP has been implicated in maintaining a healthy CNS, as PACAP knockout (KO) mice display age-related neurodegenerative signs much earlier than wildtype animals, including increased neuronal vulnerability, signs of systemic degeneration, and a heightened inflammatory state [142]. In contrast, VIP has been emphasised more for its anti-inflammatory properties [143], with evidence showing its involvement in protecting both neurons and myelin, as well as in reversing motor defects and reducing lipid peroxidation in 6-OHDA parkinsonian rats [144].

**Figure 2:**
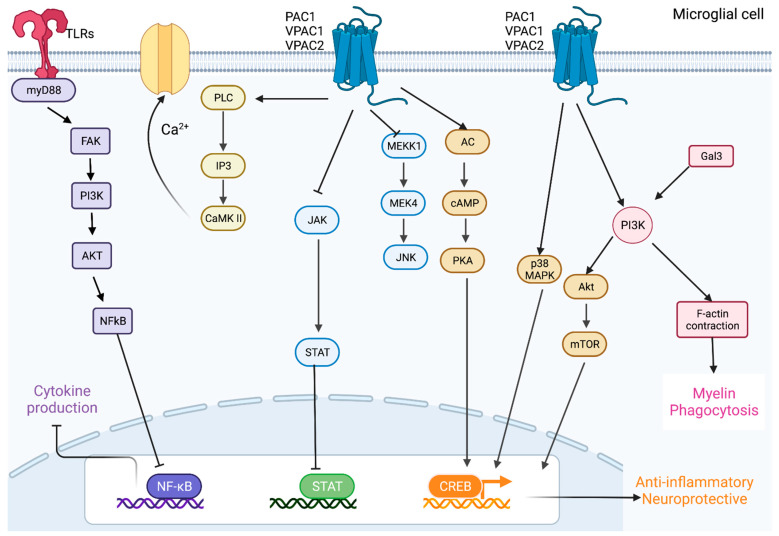
**Schematic diagram of the signalling pathways activated by PACAP and VIP in microglia**. In the figure, the molecular signalling pathways associated with microglial-mediated myelin debris phagocytosis are visualised. Briefly, myelin debris is recognised by TLRs, MerTK, TREM2, FCγR, CR3, or SRA-AI/II receptors, which either stimulates the release of pro- and anti-inflammatory cytokines, influencing myelin phagocytosis, or activates several signalling pathways involved in the remodelling and contraction of the cytoskeleton, facilitating the engulfment of myelin debris.

PACAP and VIP exert their activities by activating three G-protein-coupled receptors (GPCRs), PAC1, VPAC1, and VPAC2, each of which appears to mediate distinct biological and cell-specific functions [145]. This is of crucial importance for comprehending the activities of the PACAP/VIP system. In fact, despite all PACAP/VIP receptors recognising with high affinity both PACAP and VIP, the PAC1 receptor shows a much higher selectivity for PACAP, whereas both VPAC receptors exhibit equal high affinity for both PACAP and VIP [146]. Upon peptide binding, these receptors undergo conformational changes that allow the engagement of their intracellular domains with G proteins to either activate/inhibit a myriad of intracellular signalling cascades (Figure 2). As mentioned, VPAC1 and VPAC2 are implicated in mediating most of the immunomodulatory effects of the peptides, whereas PAC1 predominantly mediates growth/trophic factor release and other neuroprotective actions. Pathways that are commonly activated by these peptides include but are not limited to the adenylate cyclase/cAMP pathway, phospholipase C (PLC)/calcium pathway, cAMP-response element-binding protein (CREB) pathway, and G-protein-independent pathways such as PI3K and MAPK cascades [147].

### 3.1. PACAP and VIP in Multiple Sclerosis

The role of PACAP and VIP in MS has been extensively reviewed [137,141,143]. Furthermore, we recently published a review article where we discussed the beneficial effects of these peptides in ameliorating several of the neurological comorbidities of MS, outlining the potential usefulness of PACAP/VIP-based therapies as an all-in-one approach to counteract the disease by tackling distinct pathological domains [148]. However, whilst these studies have highlighted the ongoing efforts by several independent research groups in defining the proper strategy to maximise the therapeutic potential of these peptides in the context of MS and other neurodegenerative disorders, they have also emphasised the need for further investigations aimed at better characterising their contributions to central myelin repair mechanisms.

Both neuropeptides can counteract several pathogenic mechanisms triggered by MS. Exogenous treatment with either peptides reduces both histopathological and clinical scores in preclinical models of MS. Studies using PACAP KO mice have shown that the disease severity of MOG35-55-induced EAE was increased, correlating with enhanced Th1/Th17 and diminished Th2 responses [149]. Conversely, VIP KO mice were refractory to EAE-induced CNS inflammation, thus showing that immune cells failed to invade the CNS parenchyma [150]. These results provide indirect indications that PACAP and VIP are protective in the EAE model of MS. In addition, these studies have led to extensive research focusing on the roles of PACAP and VIP in T-cell function and the immune system in general [151,152,153]. 

Only a few studies using EAE (or other MS models) have investigated the potential therapeutic benefits of PACAP/VIP peptides during demyelination. Most recently, a pioneering study employing conditional PAC1 KO mice with targeted deletion of the gene *Adcyap1r1* in the retina elegantly demonstrated that gene ablation exacerbated axonal pathology and increased microglia polarisation in the retina of mice undergoing EAE, highlighting the retinoprotective role mediated by the receptor in an MS model of optic neuritis [154]. Although important, the goal of the study was to investigate the neuroprotective effects mediated by this PACAP/VIP receptor, not on myelin repair.

As discussed in Section 2.2, cuprizone-fed animals are largely used to model MS, particularly to study the demyelination occurring during MS and/or test the efficacy of drugs endowed with myelin repair properties [46]. In addition, the reversible nature of cuprizone-induced demyelination allows researchers to investigate spontaneous remyelination, a natural repair process that is impaired as the disease progresses [155]. Little work has been performed using the cuprizone model to investigate the role of PACAP and VIP in de/remyelination and neuroprotection. We have preliminary evidence showing that both peptides are able to prevent the locomotor deficits caused by a 4 week cuprizone diet (unpublished observations); therefore, it cannot be excluded that the positive effects of PACAP and VIP might be due to (1) protective effects causing increased OL and OPC survival and/or (2) enhanced myelin repair. 

### 3.2. Role of PACAP and VIP in Myelination 

Although the focus of this review paper was to discuss the involvement of PACAP and VIP in regulating microglial phagocytosis and inflammatory responses during demyelination, given the relevance to MS pathology, for completeness, we sought to introduce some of the state-of-the-art research relevant to PACAP/VIP roles in myelination.

It has been established that demyelination precedes axonal damage and neurodegeneration in MS. Therefore, strategies aimed at arresting this process and/or aimed at enhancing myelin repair mechanisms could be useful in preventing neurodegeneration and the progressive disability of MS sufferers [156]. Although we recently reiterated the importance of additional research to elucidate the exact involvement of these peptides in CNS myelination and in regulating OL functions [148], not the same can be said regarding PACAP/VIP activities on the homologous counterpart of the PNS—Schwann cells—and in peripheral neuropathies. We showed that PACAP and VIP prevent apoptosis of Schwann cells in vitro [146], and we found that PAC1 activation by PACAP promotes the proteolytic activity of these cells (critical for debris clearance) [157]. In addition, both peptides increase the expression of myelin related proteins in Schwann cell cultures [158]. More recently, our laboratory discovered that the antibiotics doxycycline and minocycline both promote similar proteolytic activity of Schwann cells as PACAP, perhaps acting as positive allosteric modulators of the PAC1 receptor [159]. Moreover, VIP and PACAP have been shown to both promote myelin gene expression and inhibit the release of pro-inflammatory cytokines by Schwann cells [160]. Schwann cells and OLs differ in embryologic origin and phenotype, and are localised in different nervous system compartments (PNS vs. CNS). However, the overlapping role between these two cell types, along with the promyelinating effects of PACAP and VIP, bodes well for future research on the possible use of PACAP/VIP analogues as boosters of myelin repair both in the PNS and in the CNS. 

## 4. Effects of PACAP and VIP in Microglia 

In the MS brain, oxidative stress contributes to demyelination and neurodegeneration in a process that involves the oxidation of proteins, lipids, and DNA, which also causes damage to mitochondria, resulting in energy deficits and further generation of reactive oxygen species (ROS) [161]. In this context, OPCs and OLs are hindered in their ability to proliferate, differentiate, and repair myelin due to a toxic nonpermissive CNS microenvironment. Microglia, as CNS scavengers, hold the potential to remove any toxic/oxidised myelin/cell debris and damaged mitochondria, contributing to the healthy microenvironment needed to allow these biological activities to occur. However, some of the mechanisms that regulate the activities of microglia, particularly phagocytosis, have not been fully investigated.

PACAP exerts essential regulatory functions on several biological activities of microglial cells, as evidenced by studies using PACAP-deficient mice [162]. In a model of drug-induced retinal injury, an intravitreal injection of PACAP was sufficient to suppress retinal neuronal loss whilst significantly increasing microglia populations and triggering an anti-inflammatory phenotype [163]. Similarly, VIP has been shown to inhibit proinflammatory mediators released by activated microglia in vitro [143]. Endogenous VIP was also shown to promote microglia proliferation and exert pro-neurogenic effects via VPAC1 activity during hippocampal neurogenesis [164]. This is further supported by evidence in preclinical models of brain trauma showing how VIP treatment prevents neuronal death by reducing the inflammatory burden caused by activated microglia [165]. These anti-inflammatory effects of VIP could also be mediated by the VPAC2 receptor, as a VPAC2 agonist, LBT-3627, reduced the subset of proinflammatory microglia and protected dopaminergic neurons in a parkinsonian rat model [166]. 

Delgado’s research group has published several studies illustrating how PACAP and VIP significantly reduce the chemotactic activity of microglia [167] and inhibit the production of proinflammatory mediators, including TNF-α, IL-1β, IL-6, and NO in LPS-stimulated microglia [168]. These studies highlighted the immunomodulatory role of these peptides, favouring the shift to anti-inflammatory phenotypes. In line with these results, in a recent study, we demonstrated that, while both PACAP and VIP attenuated LPS-induced microglial activation and cytokine inflammatory profiles, each peptide triggered a phenotypic shift towards specific microglial populations with different morphology and migratory capacity [169]. More specifically, PACAP was more efficient in restoring LPS-induced impairment of cell migration and expression of urokinase plasminogen activator (uPA) compared with VIP [169]. Proteolytic enzymes such as uPA are essential in the degradation of the extracellular matrix (ECM) and the removal of cell debris [170,171]; therefore, the PACAP-induced uPA increase in microglia may point to a role in that direction. Further investigations are warranted to address this specific question. 

In different works, rodent peritoneal macrophages have been employed to study the effects of both neuropeptides in phagocytosis. In one study, VIP increased the phagocytosis and ECM digestion of rat peritoneal macrophages [172], findings that were supported by another research that, instead, tested PACAP in mouse macrophages, with similar results [173]. More recently, Song et al. demonstrated that VIP markedly increased microglial phagocytosis via protein kinase C (PKC) signalling [174]. Moreover, in trophoblast cells, VPAC2 overexpression enhanced phagocytosis, which was associated with an anti-inflammatory microenvironment [175]. Additionally, PACAP enhanced phagocytosis of macrophages and inhibited the release of proinflammatory cytokines [176]. The indications that microglial release of anti-inflammatory mediators is paralleled by heightened phagocytic responses after PACAP or VIP treatment suggest that the peptides may trigger an anti-inflammatory phenotype in macrophages that, in turn, contributes to boosting cell-scavenging activities [177]. This theory is corroborated by additional evidence showing that the switch to an anti-inflammatory phenotype increased the engulfment of apoptotic cells [178]. Based on the above, the general idea is that PACAP and VIP may promote the phagocytic activity of both peripheral and central myeloid cells, mainly via an indirect mechanism involving the initial transition of these cells towards an anti-inflammatory state. Once transitioned, peptide-stimulated microglia cells secrete anti-inflammatory molecules that act in a paracrine manner to promote phagocytosis. However, it is also possible that the peptides activate other more direct signalling mechanisms that are independent of the switch in microglial phenotype (Figure 2), as some evidence indicates that proinflammatory signalling can also partake in the enhancement of phagocytic activities [122].

### 4.1. PACAP and VIP Promote Anti-Inflammatory Pathways in Microglia 

A great body of work has indicated that both PACAP and VIP activate anti-inflammatory signals in several in vivo and in vitro models, especially in microglia. Inhibitory effects of these peptides on the release of proinflammatory mediators such as TNF-α and inducible nitric oxide synthase (iNOS), as well as the stimulation of the anti-inflammatory cytokine IL-10, are mediated by the VPAC1 receptor via cAMP-dependent transduction pathways [179]. Additionally, both peptides have been shown to block interferon-γ (IFN-γ)-induced microglia inflammation by inhibiting the Janus kinase (JAK)/signal transducer and activator of transcription 1 (STAT1) pathway and controlling the gene expression of CD40, a critical mediator in the inflammatory cascade [180]. Furthermore, in a separate study, the same group demonstrated that both PACAP and VIP inhibit the MEKK1/MEK4/JNK pathway, another intracellular mechanism involved in microglial activation [181]. It was also found that pre-treatment of BV2 microglia with PACAP inhibited the activation of the TLR4/MyD88/NF-κB signalling pathway and decreased inflammatory cytokine levels, thereby attenuating microglial polarisation in response to hypoxic injury [182]. This same phenomenon was observed in a rat model of traumatic brain injury (TBI), where PACAP exerted neuroprotection by inhibiting secondary inflammation via the same TLR4 pathway in both microglia and neurons, resulting in reduced neuronal death, overall inflammatory burden, and improved recovery [183].

Furthermore, there is evidence that p38 MAPK activation is prevented by PACAP in microglia, and blockade of p38 MAPK activity inhibits iNOS induction [184]. In BV2 microglia, PACAP inhibition of IFN-γ-induced NO release is mediated by the increase in cAMP production, which inhibits p38 MAPK activation [185]. The structurally related peptide VIP also seems to target similar pathways in microglia, as demonstrated in an animal model of Alzheimer’s disease, where the authors showed that VIP inhibited inflammation by blocking the signalling of p38 MAPK, p42/p44 MAPK, and NF-κB intracellular cascades [186].

We showed that both peptides promote the expression of myelin-related proteins via PAC1/VPAC2 receptor activation of PI3K/Akt signalling in Schwann cells [158]. PI3K/Akt signalling is also associated with the anti-inflammatory and neuroprotective functions of PACAP in the adult brain [147,187]. Interestingly, we also revealed that the PI3K/Akt pathway is activated in a model of high-fat diet-induced brain inflammation, and metformin treatment restored both this pathway and the associated PACAP/VIP dysregulations, reducing the overall inflammatory burden [188]. Together, this suggests that the PI3K/Akt cascade is also part of the complex signalling that promotes the anti-inflammatory and protective functions in microglia. 

It is important to note that several of these pathways result in the induction of CREB, a transcription factor involved in the transcription of genes containing a cAMP-responsive element, including IL-2, IL-6, IL-10, and TNF-α [189]. As discussed earlier, these factors contribute to microglia phagocytic activity, whilst stimulating the production of anti-inflammatory factors. CREB activation also promotes the expression of proteolytic enzymes, whose release facilitates microglial phagocytic function [190]. In prior work, we showed that in Schwann cell lines, PACAP/PAC1 activation stimulated the expression of tissue plasminogen activator (tPA) in a PI3K/Akt/CREB-dependent manner to promote proteolytic activity [158], activities that mimicked brain-derived neurotrophic factor (BDNF) [158].

### 4.2. PACAP and VIP Activate Protective Pathways in Neurons and Microglia 

The acquisition of an anti-inflammatory phenotype in microglia is accompanied by the release of neurotrophic factors that promote glial and neuronal survival via the activation of antiapoptotic pathways [184].

There is a strong relationship between PACAP and VIP peptides and the expression/activity of the neurotrophic factor BDNF. We previously showed that BDNF mimics the actions of PACAP on tPA expression and activity via Akt and MAPK pathways [157], and there is additional evidence indicating that stereotactic injections of the PACAP peptide in specific CNS regions increase BDNF production [191,192]. 

Additionally, PACAP has been shown to inhibit apoptotic pathways in neuronal progenitor cells exposed to IFN-γ via a caspase 3-dependent mechanism [193]. However, in the BV2 microglial cell line, we reported that neither PACAP nor VIP reliably prevented cell loss induced by rotenone [194]. Altogether, these findings suggest that both peptides promote neuronal and, albeit less efficiently, microglial survival in response to different types of insults. There is also indication for an involvement of an indirect inhibitory effect on the apoptotic pathways, which seems to be mediated by BDNF. However, the lack of pro-survival responses in rotenone-treated microglia suggests that the protective efficacy of PACAP and VIP might depend on the type of insult/challenge to which microglial and neuronal cells are exposed to.

### 4.3. PACAP and VIP Regulate Oxidative Stress

Toxic myelin debris generated by demyelinating/degenerating neurons, in conjunction with an overactive immune response, represents two mechanisms responsible for the accumulation of ROS, the main cause of oxidative stress. Recently, several studies have begun to focus on investigating the role of PACAP and VIP in oxidative stress and mitochondrial function. Waschek and collaborators postulated that the ability of PACAP to induce mitochondria biosynthesis might provide a plausible mechanism to explain how this peptide dampens the detrimental effects of oxidative insults in neurons and perhaps other glial cells (reviewed in [137]). This idea has been corroborated by several other investigations showing that PACAP treatment attenuates mitochondria-mediated oxidative stress and neuronal apoptosis [195] and inhibits the generation of ROS [196]. Interestingly, one study demonstrated that activation of the PACAP/PAC1 axis was protective against oxidative stress-induced cell death in astrocytes, whereas VIP was devoid of any antioxidant activity in this in vitro model [197]. Conversely, in a model of ulcerative colitis, VIP reliably restored mitochondrial function, with equivalent efficacy to super oxide dismutase and dimethyl sulphide, unveiling a novel free-radical-scavenging property of VIP [198]. Such a novel function of VIP was also demonstrated in the CNS, as the peptide dose-dependently decreased the translocation of cytochrome c from mitochondria to the cytoplasm and prevented apoptosis in rat hippocampal stem cells [198]. Although there is still insufficient evidence to postulate that oxidative stress may hinder microglial phagocytosis, this needs to be taken into consideration as phagocytosis normally occurs in microenvironments where the redox balance is impaired. Therefore, it is possible that PACAP or VIP secreted by phagocytising microglia or neighbouring cells will help to mitigate the redox imbalance and aid in cell survival and debris engulfment. 

## 5. PACAP and VIP Modulate the Biological Activities of Glial Cells

Microglial cells communicate closely with other glia cells, including astrocytes and OLs, in order to maintain CNS homeostasis. The two peptides PACAP and VIP are endogenously expressed in several glial cell types, and they play regulatory functions in different biological processes in astrocytes, OLs and microglia, in both healthy and pathological conditions, such as MS. This section discusses current knowledge on the mechanisms through which PACAP and VIP regulate the activities of astrocytes and OLs and their crosstalk with microglia. 

### 5.1. Astrocytes 

Astrocytes are the most abundant glial cell type and are primarily involved in maintaining a healthy environment for neuronal signalling [199]. Activated astrocytes that release proinflammatory cytokines reduce myelination and promote axonal injury [200]. VIP, more so than PACAP, protects the white matter against excitotoxic insults and promotes axonal growth [201,202], likely through the activation of PKC survival pathways by supporting astrocytes [201]. PACAP protects astrocytes against H_2_O_2_ damage via PAC1 activation of PKA, PKC, and MAPK pathways, which collectively contribute to prevent oxidative stress and preserve mitochondrial membrane integrity [203]. These overlapping functions seen in microglia and astrocytes demonstrate that these peptides promote an overall protective response of neuroglia, which also facilitates CNS repair after injury. 

There is evidence that PAC1- and VPAC1/2-mediated activation of the PLC/calcium pathway promotes the secretion of neurotransmitters and neurohormones and may contribute to the neuromodulatory functions of the peptides [147]. It is known that PACAP and VIP can act as neuromodulators for several neurotransmitters, including dopamine, serotonin, and glutamate [204,205]. There is evidence of a strong interaction between these peptides and the dopaminergic and serotonergic systems. Pharmacological studies have shown that PACAP has neurotrophic and neuroprotective actions on dopamine and serotonin neurons [206]. Previous studies in D3R knockout (KO) mice revealed elevated hippocampal PACAP expression in the absence of the receptor, suggesting a possible link with PACAP and VIP in the processing of fear memories [207]. Moreover, the protective effect of PACAP administration correlated with increased dopamine levels [208]. In a rotenone model of PD, we revealed that buspirone, a 5HT1a agonist and D3R antagonist, alters the expression profile of these peptides throughout the brain [209]. We showed that D3Rs are expressed in microglia, and that D3R antagonism is anti-inflammatory [210]. Additionally, VPAC2 activation has been postulated to upscale astrocytic glutamate transport [211], thereby increasing neurotransmitter reuptake. Interestingly, glutamate transmission is impaired in demyelinated lesions [212]. Consequently, the effect of these peptides on astrocytes could regulate glutamate release, preventing excitotoxic insults to OLs, OPCs, and microglia. 

### 5.2. Oligodendrocytes 

The ability of PACAP and VIP to inhibit microglial production of IL-1β is a good indicator that both peptides may be effective in reinstating myelination. In fact, microglial-derived IL-1β has been shown to hinder myelin production and repair processes, whilst suppressing OPC maturation [213]. It has now been verified that the crosstalk between microglia and OLs is an essential component of the myelin repair machinery in demyelinating diseases. PACAP exerts growth factor functions on OLs and promotes the proliferation of OPCs [214]. However, its effects in configuring OLs myelinating functions is still understudied and partly controversial. In 2011, a study using PACAP KO mice found that these mice displayed a similar sequence of myelination to aged-matched wildtype mice, although the onset of myelination occurred earlier [215]. The authors concluded that endogenous PACAP exerted an inhibitory role on myelination in vivo. However, given the similar sequence of myelination seen in both genotypes and the comparable levels of myelin once mice reach adult age, it cannot be ruled out that PACAP inhibition of myelination is only a transient and developmentally conserved process that, as the same authors propose, favours neuronal maturation over myelin synthesis in critical stages of development. This hypothesis is supported by the evidence indicating that the PACAP-preferring PAC1 receptor, which is highly abundant in the CNS white matter [216], undergoes extensive alternative splicing during development to fine-tune neuronal activities [217]. Perhaps a similar PAC1-regulated myelination process, similarly to neurons, is essential for the correct maturation of the CNS and its insulating matter. 

## 6. Conclusions

Microglia elicit complex roles in the CNS of MS patients, where these cells orchestrate both acute and chronic inflammatory responses, as well as play an important role in the remyelination process of the brain [10,27,218]. In this review, we explored the role of microglia in the CNS, with special emphasis on their critical role as phagocytes of myelin debris, which, under demyelinating conditions, is a process that is necessary to promote remyelination [78,219,220]. Our deep dive into the molecular mechanisms behind myelin engulfment highlighted the intricate network of signalling cascades involved in the controlled phagocytic response by microglia to myelin debris (Figure 1). Moreover, we theorised the therapeutic potential of neuropeptides PACAP and VIP as “broad microglia modulators”, able to stimulate an anti-inflammatory microglial phenotype with increased prophagocytic potential.

Although there is still sparse evidence providing a clear/direct link between PACAP and VIP and microglial phagocytosis, we identified at least three main overlapping pathways commonly activated by microglia (during phagocytosis) and in PACAP/VIP-treated microglia. These are MyD88/NF-κB signalling, the induction of galectin-3, and PI3K/Akt signalling [158,182,221]. Additionally, the shift towards an anti-inflammatory microglial phenotype triggered by PACAP and VIP treatment promotes the secretion of prophagocytic cytokines, including IL-10 [131,222], suggestive of an indirect effect of the peptides. Lastly, throughout the review, we also explored some of the broader effects of PACAP and/or VIP as potential therapeutics in models of neurodegeneration and traumatic injury, highlighting that the pleiotropic activities and, in most cases, beneficial activities of these neuropeptides extend beyond the CNS. 

**Figure 3:**
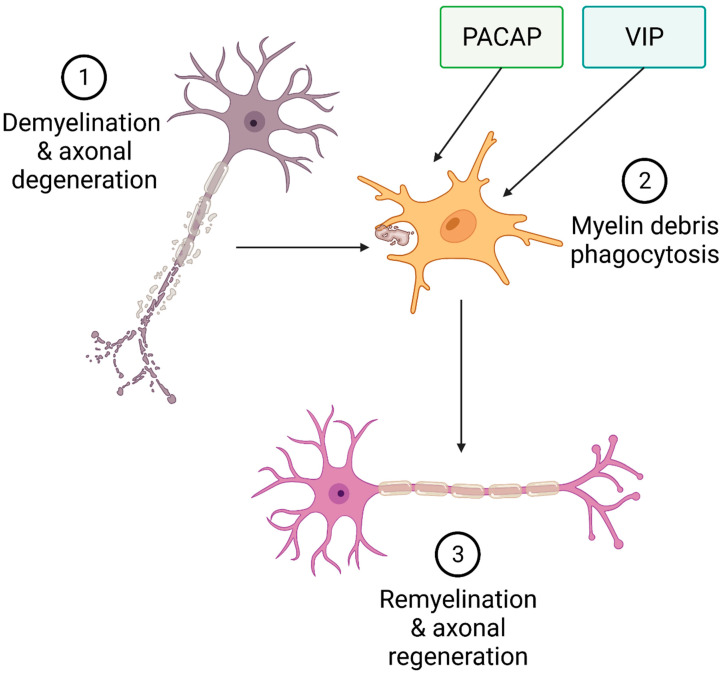
**Proposed model for PACAP and VIP prophagocytic activities in microglia**. In this simplified model, myelinated fibres of the CNS undergo immune attack, resulting in myelin degradation, axonopathy, and neuronal death (**1**). Both PACAP and VIP activate microglia to promote phagocytosis of myelin debris (**2**), creating a permissive microenvironment that allows myelin repair (**3**).

Altogether, the evidence presented here supports the hypothesis that both PACAP and VIP are potential inducers of microglial phagocytosis and could aid in stimulating the removal of myelin debris in demyelinating diseases such as MS (Figure 3).

## Data Availability

Not applicable.
